# Infrared Spectroscopy on Smoke Produced by Cauterization of Animal Tissue

**DOI:** 10.3390/s100402694

**Published:** 2010-03-26

**Authors:** Michele Gianella, Markus W. Sigrist

**Affiliations:** Laser Spectroscopy and Sensing Lab, Institute for Quantum Electronics, ETH Zurich, Schafmattstr. 16, 8093 Zurich, Switzerland; E-Mail: michele.gianella@phys.ethz.ch

**Keywords:** infrared laser spectroscopy, surgical smoke, *in vitro*, difference frequency generation, PACS, 42.62.Fi, 33.20.Ea, 87.64.km

## Abstract

In view of *in vivo* surgical smoke studies a difference-frequency-generation (DFG) laser spectrometer (spectral range 2900–3144 cm^−1^) and a Fourier-transform infrared (FTIR) spectrometer were employed for infrared absorption spectroscopy. The chemical composition of smoke produced *in vitro* with an electroknife by cauterization of different animal tissues in different atmospheres was investigated. Average concentrations derived are: water vapor (0.87%), methane (20 ppm), ethane (4.8 ppm), ethene (17 ppm), carbon monoxide (190 ppm), nitric oxide (25 ppm), nitrous oxide (40 ppm), ethyne (50 ppm) and hydrogen cyanide (25 ppm). No correlation between smoke composition and the atmosphere or the kind of cauterized tissue was found.

## 1. Introduction

Surgical smoke is a generic term that describes gases, vapors and aerosols produced in surgery with lasers, high frequency electric knives and vessel sealing devices, as well as ultrasonic scalpels. These tools are employed to cut, coagulate, ablate, dissect or fulgurate biological tissues. Heat is usually produced and (surgical) smoke develops. There is concern about the health risks posed by surgical smoke, both to the patient and to the medical staff who is exposed on a day-to-day basis [1–4]. A related issue concerns smoke produced by cooking meat, to which, e.g., kitchen personnel in restaurants is exposed daily.

So far, the biological [5,6], particulate [7–9] and chemical [9–14] compositions of surgical smoke have been studied; an excellent review is given in Ref. [15]. With respect to the chemical composition there is a general lack of quantitative data. Also of interest are possible correlations between the smoke composition and the employed tool, the kind of tissue and the insufflant gas.

As a preliminary step towards the investigation of surgical smoke produced *in vivo*, we present results on the *chemical* gaseous composition of smoke produced *in vitro* in our lab with animal meat in a specially designed cell and analyzed with a difference-frequency-generation (DFG) based mid-infrared laser spectrometer. Additionally, Fourier-transform infrared (FTIR) spectra were recorded for a few samples. For all samples a quantitative analysis was carried out with a specially developed algorithm [16]. Possible correlations between composition and insufflant as well as between composition and tissue were investigated.

## 2. Experimental Section

### 2.1. Laser Spectrometer and FTIR Spectrometer

**Figure 1.**
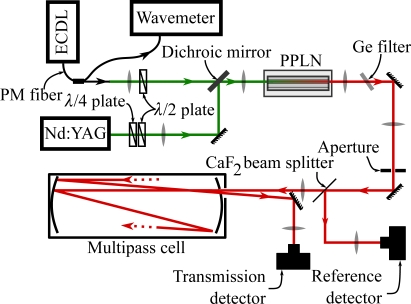
Difference-frequency-generation (DFG) based laser spectrometer used in this study. Signal beam: fiber-coupled *ECDL* (external cavity diode laser); PM fiber: polarization maintaining fiber; pump beam: *Nd:YAG* laser. λ/2 and λ/4 plate: half-wave and quarter-wave plate.

The spectrometer used in this study (Figure 1) is a difference-frequency-generation (DFG) based laser spectrometer. It has been used for several studies [16–18] but since some modifications were made it is briefly described here.

The pump laser is a diode-pumped, passively Q-switched Nd:YAG laser (InnoLight, Model M800, Germany), with a repetition rate of about 8 kHz, 6 ns pulse duration, 300 mW average power, >5 kW peak power and 1064.555 nm wavelength. The signal laser is a cw external cavity diode laser (ECDL, Santec Corp., Model TSL-210, Japan) tunable between 1520 and 1600 nm with a minimum of 5 mW of power over the entire range. The non-linear material is a periodically poled lithium niobate (PPLN) crystal, 0.5 mm thick, 10 mm wide and 50 mm long with 8 different poling periods of which only the 29.9 μm period was used in this study. It is placed in a crystal oven (Super Optronics, USA) within a Teflon housing. Quasi phase matching (QPM) is achieved by temperature tuning. The full mid-IR range of the idler beam attainable with the current signal laser (and by using both the 29.9 and 29.5 μm poling periods of the PPLN) spans from 2815 to 3144 cm^−1^ (329 cm^−1^) and could be extended by using a signal laser at wavelengths below 1520 nm. The average power of the (pulsed) idler depends on the wavelength and is typically 100–150 μW. The line width of 155 MHz is given by the pulsed Nd:YAG laser. The homemade multipass cell has been described in detail elsewhere [19]. It is vacuum-tight and can be operated up to high temperatures (*T* ≤ 720 K), although in this study we never exceeded 120 °C (393 K). The idler leaves the cell through the entry window after a number of passes that can be altered by changing the distance between the two mirrors and is then reflected into the transmission detector (Vigo Systems SA, Model PDI-2TE-4/VPDC-0.1i, Poland). The total optical path length inside the cell is 34.5 m.

The preamps of the two detectors are connected to the two input channels of an A/D card (GaGe CS14100, USA) via BNC cables. The quantity of interest is the detector signal ratio (DSR) *Q*,

(1) $$Q=\frac{\text{area}\;\text{under}\;\text{transmitted}\;\text{pulse}\;\text{signal}}{\text{area}\;\text{under}\;\text{reference}\;\text{pulse}\;\text{signal}}$$

This quantity is not yet the transmittance of the sample because the denominator only represents a part of the incident power, and because the signal of the transmission detector includes losses due to the multiple reflections in the multipass cell. Additionally, fringes caused by the uncoated BaF_2_ windows of the detectors can be observed in the DSR. To obtain the transmittance of a sample the DSR *Q*_0_ of a non-absorbing gas or of the buffer gas is needed. The transmittance *T* can then be computed via

(2) $$T=\frac{Q}{Q_{0}}$$

Spectra are recorded by setting a temperature ramp on the PPLN crystal (2 °C/min) and measuring the DSR at predefined times after an initial PPLN temperature has been crossed. Typically we record 1.5 data points per second and the temperature ramp runs from 40 °C to 173 °C. At 2 °C/min a spectrum (from 2900 to 3144 cm^−1^) takes 67 minutes to complete and consists of about 6000 points. Our data shows that this method provides better reproducibility in a considerably shorter time compared to tuning the wavelength to a desired value and then setting an appropriate PPLN temperature.

In addition to the laser spectrometer measurements, transmission spectra of a few samples in a White-type cell with 4 m of total optical path length were recorded with an FTIR spectrometer (Bruker Optics Inc., Model IFS 66v, USA).

### 2.2. Preparation of the Smoke Samples

**Figure 2.**
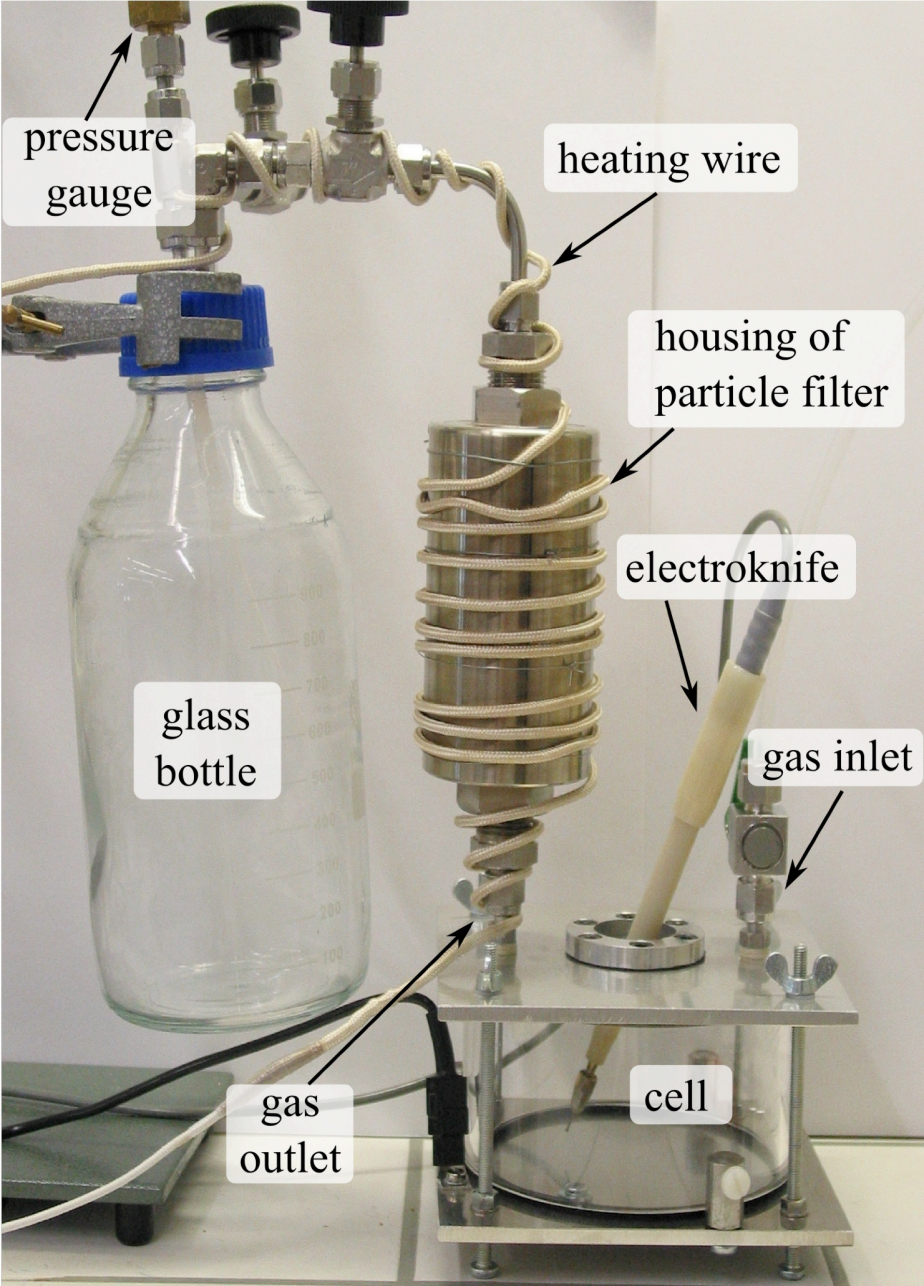
Photograph of the setup for generating and collecting *in vitro* smoke samples. The samples were prepared by cauterizing fresh animal meat with an *electroknife* inside a *cell* that allows smoke production in a specific atmosphere (e.g., CO_2_, N_2_, synthetic air).

The investigation of *in vitro* samples offers some advantages over *in vivo* samples such as: production is less time-consuming, freedom to alter any variable (e.g., change of atmosphere, type of tissue), possibility to increase concentrations by generating more smoke and so on. We designed and built a small cell that allows smoke production in a controlled atmosphere (Figure 2). The cell consists of two aluminum plates (14 × 14 × 0.5 cm), two neoprene rings (i.d. = 11 cm, o.d. = 12 cm, thickness 3 mm) and a plexi-glass cylinder (i.d. = 11 cm, o.d. = 12 cm, height 5.5 cm). A 0.5-mm thick neoprene layer is compressed between the upper aluminum plate and an aluminum ring screwed onto the plate. Two concentric holes (a 2-cm hole in the aluminum plate and a 5-mm hole in the neoprene layer) allow the insertion of a medical monopolar electroknife (Coagulasem, Ets. Dolley SA, France). All the smoke samples were produced by slowly moving the tip of the electroknife across the surface of pieces of fresh animal meat. The meat was weighed before it was put into the smoke production cell and again after smoke production to determine the loss of mass caused by the cauterization.

A gas of choice (CO_2_, N_2_, synthetic air) was pumped into the cell through the gas inlet, while the produced smoke was evacuated through the gas outlet. A micro-glass-fiber particle filter (Infiltec GmbH, housing: SL 215.401, filter elements: 25-64-30, Germany) was connected through a stainless steel tube to the outlet of the cell on one side and to a glass bottle on the other side. The housing of the particle filter and all the tubings were heated to 150 °C to prevent condensation of the smoke. The glass bottle was filled with smoke by either creating a vacuum and then aspirating the generated smoke by opening a valve, or by letting the smoke flow through the bottle for some time and then closing the valves mounted on the cap of the bottle.

Once the smoke had been stored inside the glass bottle it had to be transferred into the multipass cell. The bottle was placed into a water bath and connected to the evacuated multipass cell. The water was then heated until it boiled while small amounts of gas were transferred into the multipass cell whenever the pressure inside the bottle exceeded 1.2 bar. Once the boiling point was reached the valve between the bottle and the multipass cell was opened completely and the pressure equalized at typically 350–400 mbar. For measurements carried out at higher pressure (e.g., 930 mbar) the same gas used for the production of the smoke was added into the multipass cell directly from the gas bottle.

We investigated a total of 15 *in vitro* smoke samples all produced in a carbon dioxide (purity 2.3) atmosphere except one which was produced in nitrogen (purity 5.0), and one in synthetic air (80% nitrogen and 20% oxygen, purity unknown). The samples were produced by cauterizing fresh animal meat with a monopolar high frequency electroknife (a type of electroknife also used in human surgery). The selection of a CO_2_ atmosphere occurred in view of *in vivo* studies in laparoscopy where CO_2_ is the common insufflant gas [20]. Spectra of all samples were recorded with our DFG laser spectrometer; two of the samples were also measured with an FTIR spectrometer.

## 3. Results and Discussion

### 3.1. Quantitative Analysis

**Figure 3.**
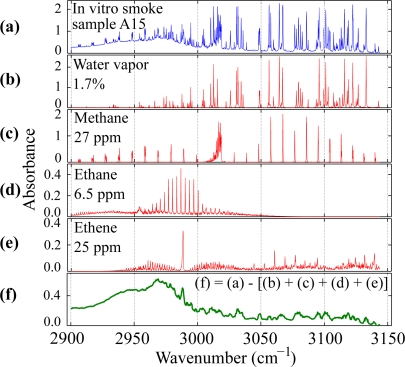
**(a)** Typical spectrum of an *in vitro* sample and its four main components: **(b)** water, **(c)** methane, **(d)** ethane and **(e)** ethene. **(f)** When the four spectra **(b)***–***(e)** are subtracted from **(a)** a residual spectrum remains.

The spectrum of a typical sample is shown in Figure 3a. Spectra of the four substances that were found in all samples are shown in Figures 3b–e. All the narrow absorption features in every measurement can be accounted for by water vapor (H_2_O), methane (CH_4_), ethane (C_2_H_6_) and ethene (C_2_H_4_) (ethylene), as can be seen in Figure 3f. The spectra in Figure 3b–e were retrieved from the PNNL database [21]. Because of the different resolution and pressure of the database spectra (0.1 cm^−1^ and 1013 mbar) and of our measurements (0.05 cm^−1^ and 900–960 mbar) some artifacts appear in Figure 3f–especially in places where several absorption lines lie closely together–due to discrepancies in the shape of the absorption lines. The concentrations for methane, ethane and ethene lie in the ppm range and water vapor is in the percent range. There is a very broad (>100 cm^−1^) absorption from 2,900 cm^−1^ to above 3,000 cm^−1^ that could not be identified unequivocally and which could be the result of the cumulative absorption of several substances or of scattering on particles smaller than 0.1 μm, if at all (since a particle filter was used). Acyclic alkanes such as pentane, hexane, *etc*., and alcohols like pentanol, hexanol, *etc*. manifest broad absorptions between 2900 and 3000 cm^−1^ that resemble the observed absorption feature. In the region above 3000 cm^−1^ there is a weaker and approximately constant absorption that is not an instrumental offset. Again, there is not enough information to associate this absorption to one or to a set of substances.

Table 1 lists for each measured sample which tissue and atmosphere were used for the smoke production, the loss of mass following cauterization, the pressure and temperature at which the spectrum was recorded, whether or not a particle filter was used, the concentrations of the four substances found in all samples, the algorithm used for the quantitative analysis (see below), and whether a Fourier-transform infrared (FTIR) spectrum was also recorded or not.

Concentrations are given in ppm = ppmV = μmol/mol. Production rates such as mol/s or mol/g would be more useful but require the total gas volume to be measured; this was not done as in many cases the smoke was produced in a continuous gas flow, of which only a fraction was sampled and measured.

The quantitative analysis of the recorded spectra was performed in most cases with the improved mix-match algorithm [16], which is based on the quantitative spectral database PNNL [21] and includes an iterative rating procedure (Alg. 1 in Table 1). The cited database consists of 360 FTIR spectra measured at ambient pressure and 25 °C: it therefore cannot be used for spectra recorded at much lower pressure (pressure broadening). For samples measured at low pressure (*p* ≤ 900 mbar) a simpler alternative to the mix-match algorithm was used (Alg. 2). It should be noted, however, that although the mix-match algorithm did not provide accurate concentrations for those samples, it still identified methane, ethane, ethene and water vapor correctly. The spectra of water, methane, ethane and ethene (at 1 ppm and 34.5 m path length) were computed with the Molspec software (Molspec III, Laser Components GmbH) using the HITRAN 2004 [22] database for *p* = 200 mbar, *T* = 100°C (for the sample A12) and *p* = 300 mbar, *T* = 120 °C (for the samples A13 and A14). The spectra were interpolated at the wavelengths at which each of the three samples was measured. The interpolated spectra were inserted into a matrix **X**, one per row, plus an additional row of zeros at the end, yielding a 5 × *k* matrix, where *k* is the number of wavelength points (typically about 6000). The principal components ***V*** and the scores **U** = X_*_V of X_*_ were computed, where X_*_ denotes **X** without the last row. The purpose of the row of zeros in **X** is to ensure that the zero spectrum belongs to the space spanned by the four principal component vectors (columns of **V**). Assuming the linear relationship **C** = **UR** between the scores **U** and the concentrations **C** (*c_ij_* = concentration of substance *j* in spectrum *i*), the matrix of regression coefficients **R** was computed in a least-square way with

(3) $$R={\left(U^{T}U\right)}^{-1}U^{T}C={\left(U^{T}U\right)}^{-1}U^{T}$$

The last equality in (3) follows from the fact that since the four spectra in X_*_ (and therefore **U**) are of pure substances, the matrix **C** is the 4 × 4 identity matrix (the units of **C** are ppm). The concentrations of water, methane, ethane and ethene of samples A12, A13 and A14 were then computed through

(4) $$C_{s}=U_{s}R=M_{s}\mathrm{VR}$$

where *s* = A12, A13 or A14, and **M**_s_ is the measured spectrum. The concentrations of samples A12, A13 and A14 have a lower accuracy–especially for ethene which has very weak absorption lines that are often in coincidence with stronger water or methane lines–compared to the samples measured at higher pressure. This is because the width of the absorption lines is smaller (typical line width at 300 mbar: 0.1 cm^−1^) but the resolution of the spectrometer was the same (0.05 cm^−1^) as for the measurements carried out at higher pressure. The purpose of the measurements carried out at higher temperature (samples A10–A14) was to increase the sensitivity to low vapor pressure species that might condensate on the inner walls of the multipass cell or on the mirrors. Every stage between the smoke production cell (Figure 2) and the multipass cell was heated in every measurement even though for only five of them the multipass cell was heated as well. No additional substance could be identified in the spectra of samples A10–A14.

Uncertainties of concentrations in Table 1 can be estimated by repeatedly measuring the same sample. The uncertainties (1 standard deviation) computed for one specific sample are: for methane, ethane and ethene about 0.5 ppm, for water about 0.025%. These values are representative for all the samples due to the similarity of their spectra and their concentrations.

### 3.2. Correlation Smoke-Tissue and Smoke-Atmosphere

Water vapor, methane, ethane and ethene are the only substances that could be identified unequivocally in each measurement and they were found in all 15 samples. Their concentrations depend on the amount of cauterized tissue (mass loss), on the volume of gas within which the smoke was diluted (dilution effect) and possibly on the atmosphere and tissue. Unfortunately, mass loss and gas volume data is not available for all measurements. We can, however, normalize the concentrations of each measurement with respect to the computed methane concentration for that spectrum and compare samples that were produced with the same tissue and atmosphere. This procedure cancels the dilution effect and might cancel the dependence of the concentrations on the mass loss, assuming all concentrations manifest the same dependence on the latter.

**Figure 4.**
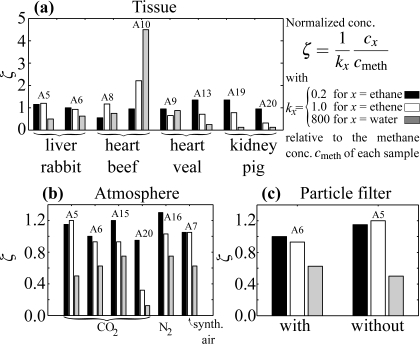
Concentrations of ethane, ethene and water vapor normalized with respect to the methane concentration in the corresponding sample. **(a)** Comparison of normalized concentrations for samples produced in carbon dioxide by cauterizing different tissues. **(b)** Normalized concentrations for samples produced in different atmospheres. **(c)** Normalized concentrations for filtered/unfiltered smoke samples. Each substance’s normalized concentration is plotted on a different scale for better visibility. To obtain the actual normalized concentration of a substance multiply ζ with the corresponding *k_x_*.

Figure 4a shows a comparison between the normalized concentrations of four pairs of smoke samples all produced in carbon dioxide. Notice that the concentration of each substance is plotted on a different scale, given by *k_x_*, for better visibility. To obtain the normalized concentration of substance x multiply the value ζ plotted on the ordinate with *k_x_* = 0.2 for *x* = ethane, 1 for *x* = ethene, 800 for *x* = water. A correlation between tissue type and smoke composition cannot be established: for example, sample A13 and A19 have very similar concentrations even though different tissue was cauterized, but sample A8 and A10 have very different concentrations although the same tissue was used. The variance of concentrations of samples produced with the same tissue is similar to the variance of concentrations of samples produced with different tissue, so that no significant tissue-smoke composition dependence can be inferred. In Figure 4b the normalized concentrations of six samples are compared, four produced in a carbon dioxide atmosphere, one in nitrogen and one in synthetic air. The atmosphere used does not seem to have any effect on the normalized concentrations of water, ethane and ethene; this is in agreement with a previous study conducted *in vitro* on animal meat [23].

The absolute concentrations of sample A16 (nitrogen atmosphere) are comparable to those of sample A6 and A15 (carbon dioxide atmosphere), see Figure 4b and Table 1. This implies that the carbon atoms in methane, ethane and ethene are not provided by the carbon dioxide atmosphere but by the tissue itself. Figure 4c shows the normalized concentrations of a sample that was filtered with a particle filter (A6) and one that was unfiltered (A5). The difference between the two samples is not significant; the filter was therefore used for most smoke samples to prevent the multipass cell and the mirrors inside it from becoming contaminated with soot.

**Figure 5.**
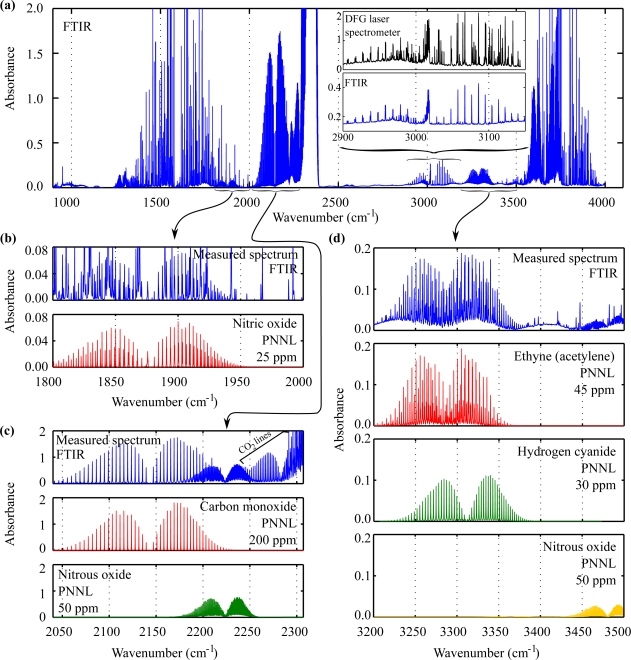
**(a)** Fourier-transform infrared (FTIR) spectrum of *in vitro* smoke sample A17 and magnification of the spectral region accessible with our DFG laser spectrometer (inset). Strong water and carbon dioxide absorptions limit the sensitivity of the spectrometer in the spectral ranges 1320–1910 cm^−1^, 2240–2380 cm^−1^ and 3530–3960 cm^−1^. **(b)** Nitric oxide (25 ppm) is visible at 1800–2000 cm^−1^. **(c)** Carbon monoxide (200 ppm) and nitrous oxide (50 ppm) can be seen at 2050–2300 cm^−1^. **(d)** Ethyne (45 ppm) and hydrogen cyanide (30 ppm) have overlapping absorption branches at 3200–3500 cm^−1^. Nitrous oxide is also visible.

As indicated in Table 1 FTIR spectra of samples A17 and A19 were recorded in addition. Aside from water, methane, ethane and ethene some additional substances that do not absorb at all or not strongly enough in the range 2900 to 3144 cm^−1^ of the DFG laser spectrometer could be identified (Figure 5): nitric oxide (NO) 25 ppm, carbon monoxide (CO) 200 ppm, nitrous oxide (N_2_O) 50 ppm, ethyne (acetylene, C_2_H_2_) 45 ppm, and hydrogen cyanide (HCN) 30 ppm. Carbon dioxide was used as atmosphere for the production of both samples, its presence is therefore obvious. Figure 5a shows the measured FTIR spectrum of sample A17 between 900 and 4000 cm^−1^. Water vapor and carbon dioxide absorptions are strong in the intervals 1320–1910 cm^−1^ (water), 2240–2380 cm^−1^ (carbon dioxide) and 3530–3960 cm^−1^ (both). The inset displays the region accessible with our DFG laser spectrometer and shows a comparison between measurements of the same sample obtained with the laser spectrometer and the FTIR spectrometer, respectively. Many water absorption lines are missing in the FTIR spectrum because of its lower resolution (0.125 cm^−1^ versus 0.05 cm^−1^ of the laser spectrometer). Figures 5b–d show magnified regions of the measured FTIR and spectra from PNNL [21] of identified components with concentrations computed with the improved mix-match algorithm [16]. The analysis of the FTIR spectrum of sample A19 yielded very similar results.

### 3.3. Detection Thresholds for Undetected Species

Across several previous studies a few hundred substances have been reported to be present in surgical smoke [9–11,15]. It is somewhat surprising that apart from the substances mentioned in the previous sections the presence of no other compound could be confirmed. In Table 2 we give the detection thresholds for some selected components that were identified in one or more previous studies and, for comparison, the detection thresholds for our DFG and FTIR spectrometer for the substances found in the present study. The presence of one or more interfering species can severely increase the detection thresholds; substances with several narrow absorption lines are only slightly affected (e.g., methane), but if large and unstructured absorption features are present the detection threshold can considerably increase. An example is toluene, which has roughly one tenth of the absorption cross-section of methane but a 330 times higher detection limit. The minimum detectable concentration (*c*_min_) for a total path length of *L* = 34.5 m (*L* = 4 m for the FTIR spectra) for a given substance *x* was determined as follows: (i) the absorbance of substance *x* at a concentration *c*_min_ is greater than or equal to *A*_min_ = 0.02 at one wavelength (at least) in the range 2900–3144 cm^−1^ (900–4000 cm^−1^ for the FTIR spectra), and (ii) the (absolute value of the) derivative of the absorbance spectrum (*i.e.*, its slope) is greater than or equal to *D*_min_ = 0.1/cm^−1^ at one wavelength (at least) in the range 2900–3144 cm^−1^ (900–4000 cm^−1^ for the FTIR spectra). *A*_min_ is the smallest measurable absorbance: it depends on the noise and the reproducibility of the measurements. By measuring a non-absorbing gas (e.g., nitrogen) several times we obtain slightly different spectra because of noise and drifts. From the variance of these spectra one can estimate *A*_min_. Since some unidentified absorption features were present in the spectra of the measured smoke samples, additional broad and unstructured absorptions are hard to notice – especially if they lie in the range 2900–3000 cm^−1^. However, if the slope of the spectrum is sufficiently large, *i.e.*, the absorption is sufficiently “sharp”, they are visible. For small molecules with narrow absorption lines condition (ii) is generally automatically fulfilled if condition (i) is true; hence, the minimum measurable absorbance *A*_min_ determines the detection threshold *c*_min_. The detection thresholds of larger molecules with broad absorptions (e.g., toluene, benzene) are quite high. The 8-hour time-averaged recommended exposure limits (REL) in Switzerland [24] are also given in Table 2. A rule of thumb for a sensor is that it should be at least a factor 10 more sensitive than the REL of the substance(s) under investigation. This condition is fulfilled for the harmless methane, ethane and ethene but not for the potentially toxic other compounds listed in Table 2.

More sensitive detection schemes or preconcentration techniques like cooling traps and thermal/solvent desorption tubes are necessary to bring the detection limits for some of the species to useful values (ppm range). Appropriate methods are currently under evaluation.

## 4. Conclusions

We present results about several *in vitro* samples of surgical smoke. Mid-infrared absorption spectra were recorded with a difference-frequency-generation (DFG) based laser spectrometer and, in a few cases, with a Fourier-transform infrared (FTIR) spectrometer. Quantitative information about the chemical composition of the samples was obtained from the absorption spectra with the improved mix-match algorithm [16]. The qualitative composition of all the samples we studied was very similar: water, methane, ethane and ethene were always found, plus additional broad absorptions that could not be identified because they are not characteristic enough. Measurements at 100 °C and 120 °C did not reveal any additional substances that were not visible at room temperature.

We investigated the relationships between smoke composition and kind of cauterized tissue on the one hand, and the atmosphere within which the smoke was produced on the other hand. In general, the variances of the concentrations in samples produced under the same conditions are comparable or larger than the variances of the concentrations of samples produced under different conditions (Figure 4). Hence, there appeared to be neither a correlation between smoke composition and atmosphere, nor between smoke composition and kind of tissue. The presence of methane, ethane and ethene in smoke produced in a nitrogen atmosphere proves that the carbon atoms originate from the tissue and not from the atmosphere.

The two FTIR spectra that were measured provided additional information about the composition of the smoke samples: ethyne, nitric oxide, nitrous oxide and hydrogen cyanide were detected with concentrations of a few tens of ppm; carbon monoxide was measured at approximately 200 ppm. Since the two samples for which the FTIR spectra were recorded were produced in a carbon dioxide atmosphere, the nitrogen atoms of nitrous and nitric oxide and of hydrogen cyanide are probably provided by the cauterized tissue (a nitrogen contamination of the carbon dioxide (purity 2.3) bottle cannot be excluded).

Our study is the first to employ exclusively infrared (laser and FTIR) spectroscopy to determine the quantitative chemical composition of surgical smoke produced *in vitro.* While the FTIR spectrometer yields a broad wavelength range, the DFG laser spectrometer enables a better spectral resolution and hence higher sensitivity yet only for a limited spectral range.

In most previous studies gas chromatography (GC), sometimes coupled with mass spectrometry (GC-MS), was the analytical tool of choice. With such techniques a few hundred chemical species have been identified (though only qualitatively) in surgical smoke (mostly *in vitro* and some *in vivo*) [10,11,13,15]. Most of them could not be confirmed here. For many, the currently used spectral range of the DFG laser spectrometer (2900–3144 cm^−1^) is not the most appropriate: although most substances of interest have some absorption in the given range (fundamental C–H stretch), the detection thresholds can be too high.

The sensitivity could be increased with a preconcentration technique like a cooling trap or with thermal or solvent desorption tubes. Without preconcentrating the samples, greater sensitivity could be achieved elsewhere in the infrared spectrum, but would limit the detectable substances to certain chemical groups (e.g., aldehydes, ketones, alkynes and so on).

Another option is the increase of power by using either a high-power cw-OPO or DFG with waveguides. Both schemes would enable the employment of more sensitive photoacoustic or cavity ringdown detection schemes, however at the cost of a large, mode-hop free tuning range which proves to be essential.

We intend to extend our study to surgical smoke produced *in vivo* in laparoscopic surgery by performing measurements in the range 2900–3144 cm^−1^ and increase sensitivity by preconcentrating the smoke samples. We are also considering the possibility of investigating surgical smoke samples around 1000 cm^−1^ (10 μm) with an external cavity quantum cascade laser (ECQCL) to address the recording of additional species.

## Figures and Tables

**Table 1.** Overview of the 15 smoke samples (A05–A20) investigated with our DFG laser spectrometer. The atmosphere (*atm.*) is either carbon dioxide, nitrogen or synthetic air (s.a.). Δ*m*: computed loss of mass. The spectra of the smoke samples were recorded at the pressure *p* and temperature *T*. The *concentrations* of methane, ethane, ethene and water vapor were computed by using one of two algorithms (*Alg.*, 1 = improved mix-match [16], 2 = PCR (see text)).

							Concentrations					
ID	Tissue	Atm.	Δ*m* mg	Filt.^a^	*p* mbar	*T* °C	CH_4_ ppm	C_2_H_6_ ppm	C_2_H_4_ ppm	H_2_O %	Alg.	FT^b^
A05	liver, rabbit	CO_2_		no	957	25	15	3.5	18	0.66	1	
A06	liver, rabbit	CO_2_		yes	956	25	14	2.8	13	0.69	1	
A07	liver, rabbit	s.a.	316.7	yes	960	25	19	4.0	20	0.99	1	
A08	heart, beef	CO_2_		yes	960	25	6.4	0.7	7.5	0.37	1	
A09	heart, veal	CO_2_	394.7	yes	960	25	11	2.1	7.1	0.74	1	
A10	heart, beef	CO_2_	769.4	yes	967	100	4.2	0.8	9.3	1.5	1	
A11	heart, pig	CO_2_		yes	900	100	13	3.0	19	1.3	1	
A12	loin, beef	CO_2_	292.6	no	200	100	12	5.5	∼15	0.35	2	
A13	heart, veal	CO_2_	269.1	yes	300	120	14	3.8	≤10	0.24	2	
A14	liver, beef	CO_2_	556.1	yes	300	120	17	6.3	≤10	1.2	2	
A15	liver, beef	CO_2_	352.8	yes	930	25	27	6.5	25	1.7	1	
A16	liver, pig	N_2_	669.0	yes	930	25	36	9.5	37	2.3	1	
A17	pig	CO_2_		yes	930	25	29	6.1	17	1.1	1	*
A19	kidney, pig	CO_2_	686.3	yes	930	25	41	11	32	0.27	1	*
A20	kidney, pig	CO_2_		yes	930	25	34	6.5	11	0.49	1	

Average							20	4.8	17	0.87		
Min.-Max.							4.2–41	0.7–11	7.1–37	0.15–2.3		

a Smoke filtered with a particle filter retaining particles ≥0.1 μm.

b A Fourier-transform infrared (FTIR) spectrum was measured for entries marked with *.

**Table 2.** Minimum measurable concentration (*c*_min_) with our DFG laser spectrometer and with the FTIR spectrometer (value in parentheses) for a few selected compounds that have been reported to be present in surgical smoke and compounds that were detected in at least one sample in this study. A dash in the minimum concentration column means that the substance has no absorption between 2900 and 3144 cm^−1^ and can therefore not be detected with our DFG laser spectrometer. Recommended exposure limits (REL, 8-hour time-average) in Switzerland [24] are also indicated.

**Substance**	***c*_min_ppm**	**REL ppm**	**Substance**	***c*_min_ppm**	**REL ppm**
**Not detected**					
Toluene	100	50	Benzene	20	0.5
p-Xylene	140	100	o-Xylene	25	100
Styrene	70	20	Ethyl benzene	40	100
Benzaldehyde	50	N/A	Benzonitrile	25	N/A
**Detected**					
Carbon monoxide	— (2.5)	30	Hydrogen cyanide	— (5.4)	1.9
Ethyne	— (4.7)	1000	Nitric oxide	— (7.5)	25
Nitrous oxide	2300 (1.2)	100	Water	120	
Methane	0.3	10000	Ethane	0.3	10000
Ethene	2	10000			

## References

[1] Freitag L., Chapman G.A., Sielczak M., Ahmed A., Russin D. (1987). Laser smoke effect on the bronchial system. Lasers Surg. Med.

[2] Gloster H.M, Roenigk R.K. (1995). Risk of acquiring human papillomavirus from the plume produced by the carbon dioxide laser in the treatment of warts. J. Am. Acad. Dermatol.

[3] Alp E., Bijl D., Bleichrodt R.P., Hansson B., Voss A. (2006). Surgical smoke and infection control. J. Hosp. Infect.

[4] Champault G., Taffinder N., Ziol M., Riskalla H., Catheline J.M.C. (1997). Cells are present in the smoke created during laparoscopic surgery. Br. J. Surg.

[5] Baggish M., Poiesz B., Joret D., Williamson P., Rebai A. (1991). Presence of human immunodeficiency virus DNA in laser smoke. Lasers Surg. Med.

[6] Hallmo P., Naess O. (1992). Laryngeal papillomatosis with papilloma virus DNA contracted by a laser surgeon. Em. Arch. Otorhinolaryngol.

[7] Weld K.J., Dryer S., Ames C.D., Cho K., Hogan C., Lee M., Biswas P., Landman J. (2007). Analysis of surgical smoke produced by various energy-based instruments and effect on laparoscopic visibility. J. Endourol.

[8] Nezhat C., Winer W.K., Nezhat F., Nezhat C., Forrest D., Reeves W.G. (1987). Smoke from laser surgery-is there a health-hazard. Lasers Surg. Med.

[9] Waesche W., Albrecht H. (1996). Investigation of the distribution of aerosoles and VOC in plume produced during laser treatment under OR conditions. Proc. SPIE.

[10] Spleiss M., Weber L., Meier T., Treffler B. (1995). Identification and quantification of selected chemicals in laser pyrolysis products of mammalian tissues. Proc. SPIE.

[11] Francke W., Fleck O., Mihalache D.L., Woellmer W. (1995). Identification of volatile compounds released from biological tissue during CO_2_ laser treatment. Proc. SPIE.

[12] Krones C.J., Conze J., Hoelzl F., Stumpf M., Klinge U., Moeller M., Dott W., Schumpelick V., Hollender J. (2007). Chemical composition of surgical smoke produced by electrocautery; harmonic scalpel and argon beaming-a short study. Eur. Sur.

[13] Al Sahaf O.S., Vega-Carrascal I., Cunningham F.O., McGrath J.P., Bloomfield F.J. (2007). Chemical composition of smoke produced by high-frequency electrosurgery. Irish J. Med. Sci.

[14] Rey J.M., Schramm. D., Hahnloser D., Marinov D., Sigrist M.W. (2008). Spectroscopic investigation of volatile compounds produced during thermal and radiofrequency bipolar cautery on porcine liver. Meas. Sci. Technol.

[15] Barrett W.L., Garber S.M. (2003). Surgical smoke-a review of the literature-Is this just a lot of hot air?. Surg. Endosc.

[16] Gianella M., Sigrist M.W. (2009). Improved algorithm for quantitative analyses of infrared spectra of multicomponent gas mixtures with unknown compositions. Appl. Spectrosc.

[17] Bartlome R., Rey J.M., Sigrist M.W. (2008). Vapor phase infrared laser spectroscopy: From gas sensing to forensic urinalysis. Anal. Chem.

[18] Bartlome R., Sigrist M.W. (2009). Laser-based human breath analysis: D/H isotope ratio increase following heavy water intake. Opt. Lett.

[19] Bartlome R., Baer M., Sigrist M.W. (2007). High-temperature multipass cell for infrared spectroscopy of heated gases and vapors. Rev. Sci. Instrum.

[20] Menes T., Spivak H. (2000). Laparoscopy-Searching for the proper insufflation gas. Surg. Endosc.

[21] Sharpe S.W., Johnson T.J., Sams R.L., Chu P.M., Rhoderick G.C., Johnson P.A. (2004). Gas-phase databases for quantitative infrared spectroscopy. Appl. Spectrosc.

[22] Rothman L.S., Jacquemart D., Barbe A, Benner D.C., Birk M, Brown L.R., Carleer M.R., Chackerian C., Chance K., Coudert L.H., Dana V., Devi V.M., Flaud J.M., Gamache R.R., Goldman A., Hartmann J.M., Jucks K.W., Maki A.G., Mandin J.Y., Massie S.T., Orphal J., Perrin A., Rinsland C.P., Smith M.A.H, Tennyson J., Tolchenov R.N., Toth R.A., Vander Auwera J., Varanasi P., Wagner G. (2005). The HITRAN 2004 molecular spectroscopic database. J. Quant. Spectrosc. Radiat. Transfer.

[23] Hensman C., Baty D., Willis R.G., Cuschieri A. (1998). Chemical composition of smoke produced by high-frequency electrosurgery in a closed gaseous environment-An in vitro study. Surg. Endosc.

[24] Suva (2009). Grenzwerte am Arbeitsplatz.

